# Case Report: A pediatric case of the clear-cell variant of mucoepidermoid carcinoma in the palate harboring *MAML2* gene rearrangement

**DOI:** 10.3389/fped.2025.1600823

**Published:** 2025-07-03

**Authors:** Hengkun Wang, Xiaoya Wang

**Affiliations:** Department of Stomatology, Weihai Municipal Hospital, Cheeloo College of Medicine, Shandong University, Weihai, China

**Keywords:** mucoepidermoid carcinoma, clear-cell variant, *MAML2* gene rearrangements, pediatric case report, palate

## Abstract

The clear-cell variant of mucoepidermoid carcinoma (MEC) is a rare subtype, with pediatric cases being extremely rare. A 13-year-old girl presented with a lesion on the right palate, which had been noted for 3 months. The patient reported pain, rupture, and bleeding of the tumor for nearly a month. Computed tomography scans revealed a quasi-circular soft tissue mass on the right hard palate. Microscopically, the tumor cells showed predominant clear cells and scattered mucous cells. The tumor cells were positive for cytokeratin (CK), CK7, CK5/6, epithelial membrane antigen, P63, and P40. A rearrangement of mastermind-like transcriptional coactivator 2 (*MAML2*) (11q21) gene was identified in the tumor cells by fluorescence *in situ* hybridization. The histological features supported a diagnosis of clear-cell variant of MEC, medium grade, with a tumor stage of pT1N0M0. The patient underwent a complete excision of the palatal mass followed by superficial bone removal. After surgery, the patient recovered well and was recurrence-free at the 1-year follow-up. Based on repeated pathological evaluations, we report this rare pediatric case of a clear-cell variant of MEC of the palate. Only surgical resection resulted in a favorable outcome.

## Introduction

Mucoepidermoid carcinoma (MEC) is the most common malignancy of the salivary glands, occurring in both major and minor salivary glands (1). MEC is typically composed of a mixture of mucin-producing cells, epithelioid cells, and intermediate cells in varying proportions (2). Although the classic form of MEC is mainly composed of mucin-producing cells, a rare subtype—known as the clear-cell variant of mucinous adenocarcinoma (cMEC)—is characterized by a predominance of clear cells. cMEC is especially rare in children (3).

Chromosome t (11; 19) (q21; p13) translocation encoding CREB-regulated transcription coactivator 1 (*CRTC1*)-mastermind-like transcriptional coactivator 2 (*MAML2*) gene fusion is the most important molecular genetic change in MEC (4). More than half of MEC harbors this gene fusion (4). In addition, *MAML2* gene fusion is more common in low-to-intermediate-grade MEC, suggesting a low risk of tumor recurrence and metastasis, as well as a favorable prognosis. In diagnostically challenging cases and various histological subtypes, the highly specific *MAML2* gene rearrangement should be used as a diagnostic tool for histopathology (5).

Herein, we report a case of a 13-year-old girl diagnosed with cMEC in the palate and harboring a *MAML2* gene rearrangement. In addition, we reviewed the literature on cMEC and discussed its rarity, diagnostic essentials, treatment, and prognosis.

## Case report

A 13-year-old girl presented with a lesion on the right palate that had been noted for 3 months. The patient reported pain, rupture, and bleeding of the tumor for nearly a month. Her past medical history was unremarkable, and her growth and development had been normal. The patient did not report her family medical history when admitted to the hospital.

**Figure 1:**
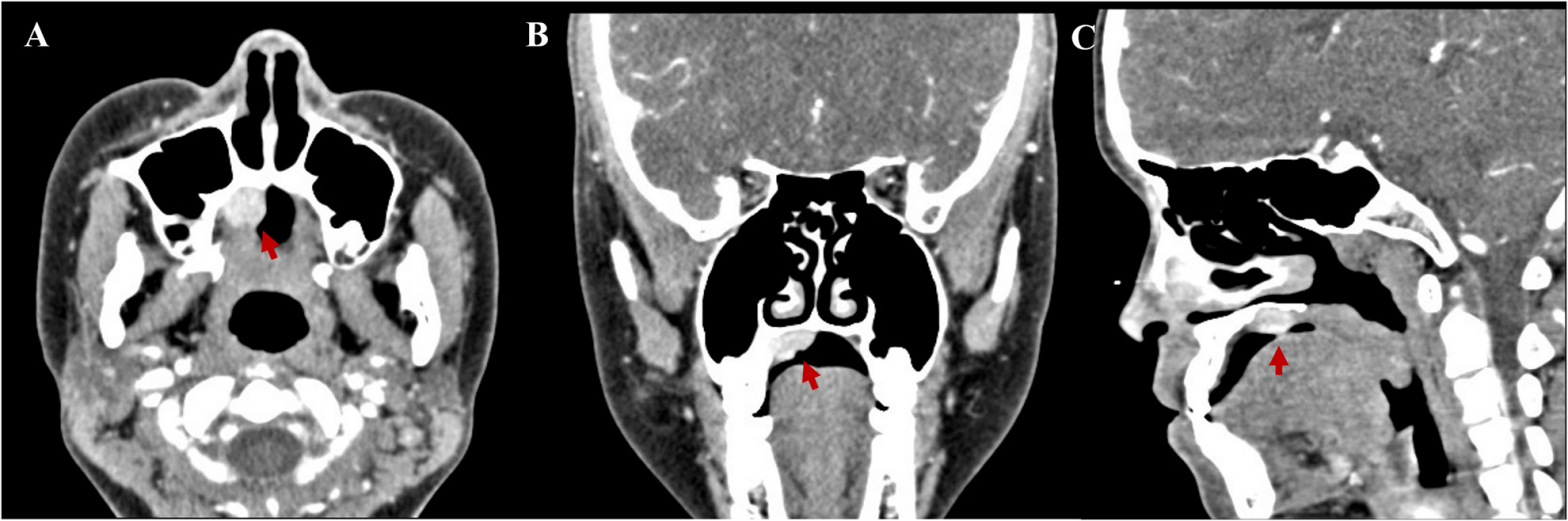
Contrast-enhanced computed tomography scans revealed a quasi-circular soft tissue mass of the right hard palate 1.5 cm × 1.2 cm × 1.0 cm in size. **(A)** Axial, **(B)** coronal, and **(C)** sagittal.

Contrast-enhanced computed tomography scans revealed a quasi-circular soft tissue mass of the right hard palate 1.5 cm × 1.2 cm × 1.0 cm in size. The mass was an enhancing nodular component, with probable bone remodeling but no definite bone invasion (Figure 1).

**Figure 2:**
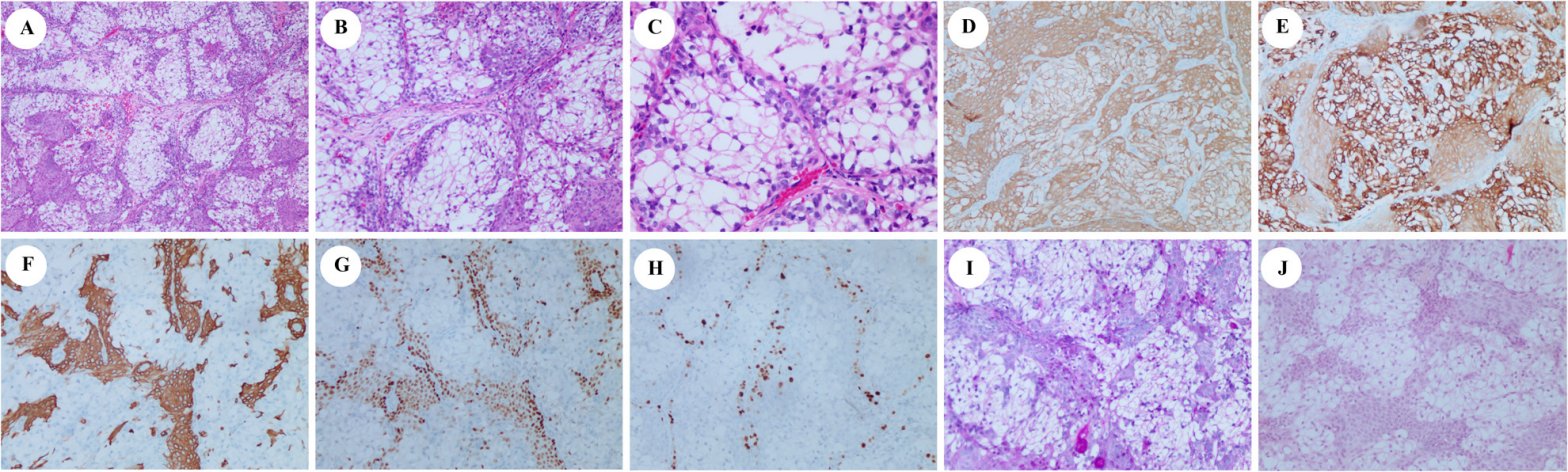
Hematoxylin and eosin (H&E), immunohistochemical, and specific staining of biopsy. H&E (**A**, ×40; **B**, ×100; and **C**, ×200). Immunohistochemistry showing the tumor cells to be positive for CK (**D**, ×100), cytokeratin 7 (**E**, ×100), cytokeratin 5/6 (**F**, ×100), and P63 (**G**, ×100). Staining for nuclear protein Ki-67 highlighted up to 10% of the intermediate cells (**H**, ×100). PAS and mucicarmine staining were slightly positive (**I** and **J**, ×100).

After a multidisciplinary team discussion, the patient underwent surgical excision of the right palate mass. Intraoperative frozen section analysis confirmed negative margins. Pathological examination revealed a tumor involving both the hard and soft palate, with an intact capsule and a smooth underlying bone surface. Microscopically, the tumor was composed of lobular and nested arrangements of clear cells and squamous cells, with scattered mucous cells observed locally. These features were consistent with a diagnosis of clear-cell mucoepidermoid carcinoma (Figures 2A–C). The tumor cells tested positive for cytokeratin (CK) (Figure 2D), CK7 (Figure 2E), CK5/6 (Figure 2F), epithelial membrane antigen (EMA), P63 (Figure 2G), and P40 (ΔNp63 proteins). Staining for nuclear protein Ki-67 highlighted up to 10% of the intermediate cells (Figure 2H). The DNA mismatch repair (MMR) protein expression, namely MLH1, PMS2, MSH2, and MSH6, was retained. The tumor cells were negative for calponin, carcinoembryonic antigen (CEA), and androgen receptor (AR). Special staining with periodic acid-Schiff (PAS) (Figure 2I), mucicarmine (Figure 2J), and Alcian blue (AB) showed weak positivity.

To further characterize the MEC, fluorescence *in situ* hybridization (FISH) analysis was performed using a laboratory-developed dual-color break-apart probe targeting the *MAML2* (11q21) gene. *MAML2* rearrangement was identified in the tumor cells using FISH. Additional FISH analysis using a similarly designed probe for *EWSR1* was negative. The histological features supported a diagnosis of clear-cell variant of MEC, intermediate grade, according to the WHO pathological grading criteria. The tumor was staged as pT1N0M0.

All margins, including those from the separately submitted tumor bed, were free of carcinoma. The patient recovered well postoperatively and remains recurrence-free at the 1-year follow-up.

## Discussion

MEC can occur at any age, but it most commonly presents in middle-aged and older adults. Approximately 64% of MEC cases are diagnosed in individuals aged 40–50 years (1). cMEC is a rare subtype of MEC. In their series, Yang et al. reported that cMEC accounted for 4.7% of all salivary gland MECs, with a predilection for minor salivary glands, particularly the palate (6). Although cMEC has been described in adults—with an average age of onset of 36.4 ± 15.3 years—pediatric cases are extremely rare (7). This case report presents a rare instance of cMEC of the palate with *MAML2* rearrangement in a 13-year-old girl.

MEC is mainly composed of three types of cells: mucin-producing cells, epithelioid cells, and intermediate cells. In addition, some rare cell types may be present, such as columnar cells and clear cells. When clear cells predominate over other cell types, the tumor is referred to as cMEC (7). Although the diagnosis of conventional MEC is straightforward, the presence of clear cells can complicate histopathological interpretation (8). It becomes extremely important to distinguish cMEC from other clear-cell lesions, as follows:

(1) Clear-cell myoepithelial carcinoma (CC-MC): like cMEC, CC-MC is predominantly composed of clear cells. However, CC-MC lacks mucinous cells and shows immunohistochemical positivity for myoepithelial markers.
(2) (Clear-cell carcinoma (CCC): CCC consists of uniform polygonal cells with clear cytoplasm and variable size. Its immunophenotype may resemble mucoepidermoid carcinoma, with strong cytokeratin and P63 positivity. However, CCC lacks mucinous cells, is negative for AB staining, and over 80% of cases exhibit an *EWSR1-CREM* gene fusion.
(3) Metastatic renal clear-cell carcinoma: this carcinoma also lacks mucinous cells and is typically negative for CK7 and P63 on immunohistochemistry. It does not exhibit *MAML2* rearrangement, which helps differentiate it from cMEC.

Additional differential diagnoses include secretory carcinoma, pleomorphic adenoma, acinic cell carcinoma, epithelial–myoepithelial carcinoma, and odontogenic clear-cell carcinoma (9). The diagnostic criteria for cMEC include (1) compared with other cell types, clear cells are the main component of tumors; (2) presence of mucus-producing cells; and (3) absence of clear-cell carcinoma metastases of the kidney or thyroid (7).

FISH detection of *MAML2* gene rearrangement is helpful in the diagnosis of MEC variants (5). Fujimaki et al. were the first to identify the *CRTC1-MAML2* fusion gene, thereby confirming the diagnosis of the eosinophilic variant of MEC (10). *MAML2* rearrangement can also be used to diagnose other MEC subtypes, such as the ciliary variant and Warthin neoplastic variant (11, 12). In pediatric MEC series, the incidence of *MAML2* gene rearrangement can be as high as 100% (13). In addition, studies have shown that the survival rate of *MAML2* gene rearrangement in MEC patients is significantly higher than that of unfused patients (5). Compared with negative cases, MEC patients with *MAML2* gene rearrangement tend to exhibit more favorable clinicopathological features, including younger age at diagnosis, smaller tumor size, lower frequency of lymph node metastasis, lower clinical stage and histological grade, and longer overall and disease-free survival (5).

Salivary gland malignancies have become increasingly common in clinical practice. Due to their diverse histopathological subtypes and the complex anatomy of the salivary glands, surgical resection remains the preferred treatment modality (13). For low-grade MEC arising in the minor salivary glands, wide local excision with clear surgical margins is typically sufficient. Previous literature (14) indicates that pediatric MECs are localized and rarely have local expansion or regional metastasis. Furthermore, pediatric tumors are more likely to be well or moderately differentiated compared to those in adults. Therefore, in this case, the patient underwent complete excision of the palatal mass, along with preventive partial bone contouring despite the absence of definite bone invasion, to ensure complete removal. For high-grade, unresectable, or recurrent MECs, postoperative radiotherapy should be used. However, radiotherapy is rarely used in children.

Recent studies suggest that targeted inhibition of the *EGFR* pathway using small-molecule *EGFR* inhibitors may offer a new systemic treatment option for MEC patients with *CRTC1-MAML2* translocations (15). However, there is still a long way to go.

The prognosis of cMEC is influenced by several factors, including tumor stage, site, pathological grade, and completion of surgery (16, 17). Compared with tumor node metastasis (TNM) stage, pathological grade is not only an independent prognostic factor of MEC but also has a greater influence on biological behavior, cervical lymph node metastasis, and prognosis of MEC. Low-grade MECs generally have an excellent prognosis, while high-grade tumors are associated with significant therapeutic challenges and poorer survival outcomes. Accurate grading and multimodal therapy are critical for optimizing outcomes.

In pediatric MEC cases, tumors are more frequently of low to intermediate histopathologic grade, which is associated with favorable outcomes (13). In the present case, the tumor was classified as a medium-grade MEC, and no recurrence was observed at the 1-year follow-up.

## Data Availability

The original contributions presented in the study are included in the article/Supplementary Material, further inquiries can be directed to the corresponding author.
